# Depression, Environmental Reward, Coping Motives and Alcohol Consumption During the COVID-19 Pandemic

**DOI:** 10.3389/fpsyt.2020.574676

**Published:** 2020-10-30

**Authors:** Matthew D. McPhee, Matthew T. Keough, Samantha Rundle, Laura M. Heath, Jeffrey D. Wardell, Christian S. Hendershot

**Affiliations:** ^1^Department of Psychology, University of Toronto, Toronto, ON, Canada; ^2^Department of Psychology, York University, Toronto, ON, Canada; ^3^Institute for Mental Health Policy Research and Campbell Family Mental Health Research Institute, Centre for Addiction and Mental Health, Toronto, ON, Canada; ^4^Department of Psychiatry, University of Toronto, Toronto, ON, Canada; ^5^Department of Pharmacology and Toxicology, University of Toronto, Toronto, ON, Canada

**Keywords:** COVID-19, SARS-CoV-2, social distancing, alcohol, mental health, stress, depression

## Abstract

**Background:** Increases in the incidence of psychological distress and alcohol use during the COVID-19 pandemic have been predicted. Behavioral theories of depression and alcohol self-medication theories suggest that greater social/environmental constraints and increased psychological distress during COVID-19 could result in increases in depression and drinking to cope with negative affect. The current study had two goals: (1) to examine self-reported changes in alcohol use and related outcomes after the introduction of COVID-19 social distancing requirements, and; (2) to test hypothesized mediation models to explain individual differences in self-reported changes in depression and alcohol use during the early weeks of the COVID-19 pandemic.

**Methods:** Participants (*n* = 833) were U.S. residents recruited for participation in a single online survey. The cross-sectional survey included questions assessing environmental reward, depression, COVID-19-related distress, drinking motives, and alcohol use outcomes. Outcomes were assessed via retrospective self-report for two timeframes in the single survey: the 30 days prior to state-mandated social distancing (“pre-social-distancing”), and the 30 days after the start of state-mandated social distancing (“post-social-distancing”).

**Results:** Depression severity, coping motives, and some indices of alcohol consumption (e.g., frequency of binge drinking, and frequency of solitary drinking) were significantly greater post-social-distancing relative to pre-social-distancing. Conversely, environmental reward and other drinking motives (social, enhancement, and conformity) were significantly lower post-social distancing compared to pre-social-distancing. Behavioral economic indices (alcohol demand) were variable with regard to change. Mediation analyses suggested a significant indirect effect of reduced environmental reward with drinking quantity/frequency via increased depressive symptoms and coping motives, and a significant indirect effect of COVID-related distress with alcohol quantity/frequency via coping motives for drinking.

**Discussion:** Results provide early cross-sectional evidence regarding the relation of environmental reward, depression, and COVID-19-related psychological distress with alcohol consumption and coping motives during the early weeks of the COVID-19 pandemic. Results are largely consistent with predictions from behavioral theories of depression and alcohol self-medication frameworks. Future research is needed to study prospective associations among these outcomes.

## Introduction

In the first 8 months of the COVID-19 pandemic, there have been over 27 million confirmed and presumptive cases of the COVID-19 infection globally (1). Attempts to curtail the spread of the virus have included localized approaches (e.g., contact tracing, quarantine) and large-scale population directives [e.g., social distancing and shelter-in-place requirements; (2)]. Given the broad socioeconomic and health impacts of the pandemic, increased incidence of psychological distress and mental health disorders are among the anticipated consequences of the COVID-19 pandemic [e.g., (3–5)]. Past evidence that societal crises (e.g., economic recessions; natural disasters) were followed by increases in mental health and substance use problems (6), and preliminary evidence of elevated levels of depression and anxiety during the COVID-19 pandemic [e.g., (7–9)], have led to calls for research to evaluate mental health outcomes during the COVID-19 pandemic.

Initial data are consistent with potential increases in alcohol consumption during the COVID-19 pandemic. For example, increased alcohol sales [e.g., (10)], elevated rates of harmful alcohol use in COVID-19 epicenters [e.g., (11)], and altered patterns of alcohol consumption [e.g., based on remote breath alcohol concentration data; (12)] have been reported. The Canadian Centre on Substance Use and Addiction (CCSA) reported that ~1 in 5 individuals who consume alcohol reported increases in alcohol consumption relative to the period prior to the pandemic, although the majority did not report an increase in alcohol consumption (13). These findings are consistent with predictions that circumstances surrounding the pandemic may lead to increases in consumption for some people, but no change or decreases for others (4), making it important to understand factors coinciding with increases in consumption.

Of numerous contextual factors that could increase risk for alcohol use during the pandemic, changes in psychological distress and mental health symptoms are important considerations. The unprecedented consequences of COVID-19, including widespread unemployment and lost income, health-related concerns, and mandated social isolation are likely risk factors for increases in depression and other forms of psychological distress among the general population. Behavioral theories of depression posit that reductions in access to environmental/social rewards, and/or increases in reward-limiting stimuli (i.e., environmental suppressors) predict risk for depression (14, 15). Measures designed to assess access to environmental reward have been developed, and evidence supports the relation between diminished environmental reward and elevated severity of depression [e.g., (16–19)]. By design, population-based approaches to virus control have imposed significant environmental and contextual constraints for large portions of the population, resulting in widespread changes to daily routines and social interactions. By way of constraining daily routines and reducing access to typical sources of social or environmental reinforcement, strict social distancing measures may increase the risk for psychological distress and/or depressive symptoms for some individuals.

Stress and negative affect are primary risk factors for increases in alcohol consumption among drinkers, and for relapse among those who have cut down or quit drinking (20). Increases in negative affect, including depression symptoms and/or generalized distress in response to challenges surrounding the pandemic, might lead to increases in alcohol consumption. As a result, some have predicted a drastic increase in alcohol relapse among vulnerable populations (10). It follows that environmental constraints related to social distancing measures might indirectly result in increased alcohol consumption, by way of increases in depression or psychological distress. Perhaps consistent with these predictions, research during the SARS epidemic found that almost one third (31.2%) of individuals quarantined had positive screens for depression (21), and among hospital employees, alcohol use disorder symptoms were positively associated with having been quarantined and working in a high-risk location (22).

Additional factors influencing drinking context or drinking opportunities could have implications for the incidence of unhealthy alcohol consumption during the COVID-19 pandemic. Solitary drinking (i.e., use of alcohol alone vs. in social contexts) is positively associated with greater incidence of alcohol-related problems (23, 24). Notably, frequency of solitary drinking (compared to drinking in social contexts) is positively predicted by severity of depressive symptoms (25). To the extent that environmental constraints may limit social drinking opportunities and increase depression symptoms, solitary drinking is likely to increase under social distancing conditions. Additionally, changes in drinking contexts (e.g., bar closures) may call for studying alternative indices of alcohol motivation, such as alcohol demand. Alcohol demand refers to the reinforcing potential of alcohol based on hypothetical resources (e.g., economic) that an individual would allocate to obtain alcohol (26). Greater alcohol demand is associated with alcohol-related problems and alcohol consumption (27, 28). Importantly, dynamic changes in demand have been observed in response to stress manipulations (29), and alcohol demand in solitary contexts predict problems associated with alcohol use beyond alcohol demand in social contexts (30). Together, these results suggest the importance of considering change in alcohol demand as an outcome during the COVID-19 pandemic.

Drinking for negative reinforcement reasons (i.e., to reduce negative affect) plays a central role in stress-related alcohol use, and is associated with significantly increased risk for alcohol problems (31). According to the Self-Medication Hypothesis (32, 33) drinking to cope with negative affect (i.e., coping motives) is a critical mediator between situational increases in negative affect and subsequent increases in alcohol use and associated problems. The self-medication hypothesis has also been used to explain the relationship between depression and alcohol use/problems [reviewed in (34)]. Evidence further suggests a mediating role of coping motives in the association of peritraumatic distress and alcohol-related problems [e.g., (35)]. While coping motives are central to the self-medication hypothesis, other domains of drinking motives include enhancement motives (i.e., drinking to enhance positive mood), social motives (e.g., affiliation with peers) and conformity motives [e.g., peer pressure; (36, 37)]. Notably, coping motives uniquely predict heavier drinking and related alcohol problems when controlling for other domains (31, 38).

While motives for alcohol consumption are often studied as static phenomena and assessed at one point in time, some studies suggest that drinking motives are subject to dynamic change [e.g., (39, 40)]. As a consequence of social (e.g., reduced interpersonal contact) and environmental (e.g., closure of public drinking venues) changes associated with the COVID-19 pandemic, changes in specific reasons for drinking are likely to occur, at least for some individuals. For instance, if social distancing requirements constrain environmental reward, increased psychological distress or depression [e.g., (41, 42)] might result in escalations in coping motives for drinking and ultimately increased alcohol use. Similarly, increased severity of fear and anxiety specifically related to COVID-19 might predict escalations in negative reinforcement drinking, consistent with the self-medication hypothesis and with past research [e.g., (43)].

Evidence from other public health crises supports these possibilities. Following the 2003 SARS outbreak, Maunder et al. (44) found that maladaptive coping was associated with self-reported increases in alcohol use among health-care workers. Additionally, in hospital employees, endorsement of using alcohol to cope with the SARS outbreak was positively related to alcohol use disorder symptoms (22). This research is limited, however, to samples directly impacted by the disease (e.g., healthcare workers, those in quarantine) and there is a paucity of research in general samples. Of note, early research published in the COVID-19 pandemic has also highlighted differences in psychological response to the pandemic associated with race. For example, Fitzpatrick et al. (45) found greater levels of COVID-19 related fear in Asian and Hispanic participants, relative to their counterparts. The psychological impact of the pandemic on non-majority groups is potentially further exacerbated by pre-existing disparities in mental health, disproportionate impact of the virus on minority groups, and discrimination (46, 47). Information on changes in psychological distress and related outcomes (e.g., depression, substance use) during the COVID-19 pandemic, and their association with race, may be used to direct intervention efforts in this and future public health crises.

The current study had two primary aims. First, following recommendations to study changes in substance use and associated risk factors during the COVID-19 pandemic (4), we aimed to assess self-reported differences in mood, environmental reward, drinking motives, and alcohol outcomes (e.g., quantity/frequency, solitary drinking; alcohol demand) in the period immediately preceding widespread social distancing measures, as compared to the period when these measures were in place. Exploratory analyses also examined whether any of these outcomes differed as a function of self-identified racial group. The second aim was to examine perceived changes in coping motives and depression symptoms as accounting for the relation between perceived change in environmental reward and psychological distress with alcohol consumption during the COVID-19 pandemic. A cross-sectional design using a single online survey assessment was employed to test these aims. Based on self-medication theory (32) and behavioral theories of depression [e.g., (15)], two primary hypotheses were tested. First, we predicted that individual differences in environmental reward during COVID-19 would predict severity of depressive symptoms, which would in turn predict coping motives and alcohol consumption. Second, we predicted that COVID-19-related psychological distress would predict greater endorsement of coping motives, which would in turn predict greater quantity/frequency of alcohol consumption.

## Methods

### Participants

Participants were U.S. residents recruited from Amazon's Mechanical Turk (MTurk) between May 12, 2020 and May 23, 2020. A total of 1,854 individuals were screened for participation. Potential participants viewed a description of the survey before electing to participate. Interested participants followed a link from MTurk to an external survey on the Qualtrics platform. Although there has been debate as to the quality of data collected from MTurk participants, past research has documented that it is both a reliable and valid platform for data collection for both the general public population (48–50) as well as those with past history of substance use disorders (51). Participants were first screened for eligibility and, if eligible, were provided an information page and asked to confirm or decline participation. After screening for eligibility and data quality (see below), a total of 833 participants were retained for analysis.

Inclusion criteria for the study included: (a) self-reported age 21+ years (b) self-reported proficiency in reading and comprehending English; (c) current state of residence with implemented mandatory social distancing procedures, and; (d) self-reported consumption of alcohol on >1 occasions per month, on average, in the past year. Exclusion criteria for the study included a reported history of COVID-19 infection in the 90 days preceding the assessment (to mitigate the effects of COVID-19 infection on alcohol consumption patterns). Additionally, participants residing in states with no mandatory social distancing (e.g., shelter-in-place or equivalent) protocol at the time of data collection were excluded from recruitment; this information was obtained from respective state government websites. The following states were excluded from recruitment: Arkansas, Iowa, Nebraska, North Dakota, Oklahoma, South Dakota, Utah, and Wyoming.

### Procedures

Eligible participants were asked to complete a brief survey (duration: ~20–30 min) that contained three distinct sets of items. The first set of items queried demographic characteristics, past-year drinking history, and psychological distress (including emotional and physiological reactions) attributed to COVID-19. The first set also included the Centers for Disease Control and Prevention definition of social distancing to ensure a standardized operational definition across all participants. Participants then proceeded to the second set of questionnaires that assessed drinking motives, alcohol use and related outcomes, depressive symptoms, and environmental reward. Before starting the second set of questions, participants were provided with specific instructions to anchor their replies to the 30 days immediately preceding the start date of state-mandated shelter-in-place (or equivalent) protocol: “In the one-month period prior to the start of the state-mandated shelter in place protocol…” Therefore, the second set of questions provided data on the outcomes of interest pre-social-distancing. Survey timeframes were individualized based on the individual's current state of residence; start dates for social distancing orders (obtained from State Government websites) were piped in to the participant's survey based on their current residence. To standardize instruction sets, the actual start date and timeframe instruction were repeated at the start of each question.

After completing the second set of items, participants proceeded to the third set of questionnaires. The items included in the third set were identical to those provided in the second set. However, before starting the third set of questions, participants were provided with specific instructions to anchor their replies to the 30 days immediately following the start of the state-mandated shelter-in-place (or equivalent): “In the 30 days immediately after the start of the state-mandated shelter-in-place protocol”. Consequently, the third set of items provided data on the outcomes of interest post-social-distancing. Because some states were in the process of ‘re-opening’ at (or soon after) the start of data collection, it was important to anchor responses to the 30-day period after the start of the mandate, rather than the past 30 days.

Five attention-check questions were interspersed throughout the survey as a means of detecting random responding. Additionally, two questions appeared at the end of the survey asking the participant to confirm that they: (1) answered the questions honestly, and (2) paid attention to the questions. These attention checks have been utilized in past research completed via MTurk (49, 50). Participant data were excluded if the participant incorrectly responded to >1 attention checks, in order to control for random responding. Upon completion of the Qualtrics survey, participants were compensated $2.50 (USD), which is comparable to the recommended $2/hour rate (52). Upon completion of the survey, participants were granted a custom qualification within MTurk that restricted them from completing the survey more than once.

### Measures

#### Alcohol Use Disorders Identification Test (AUDIT)

The AUDIT is a 10-item scale assessing hazardous alcohol use, symptoms of dependence, and harmful alcohol use in the past year (53). Seven of the ten items are scored on a 4-point scale (response options differ by question structure). The remaining three items are scored on a 3-point scale. A systematic review (54) identified numerous studies that supported sound psychometric properties of the AUDIT, including test-retest reliabilities of 0.6 to 0.84 and an average Cronbach's alpha of 0.80. Internal consistency in the current sample was 0.89. Because total AUDIT score was included as a descriptor for the sample characteristics, AUDIT scores were not anchored to the aforementioned time intervals.

#### Modified Peritraumatic Distress Inventory (PDI)

The PDI is a 13-item scale assessing peritraumatic distress, defined as the emotional and physiological distress experienced by an individual after a traumatic event (55). Items on the scale (e.g., “I felt helpless to do more”) were scored on a 5-point scale from 1 (*not at all true*) to 5 (*extremely true*). The original PDI instructions were altered to specifically capture distress attributed to COVID-19 (e.g., “Please rate the extent to which you have experienced each of the following items during (or immediately after) the COVID-19 pandemic.”). Although exposure to stress surrounding COVID-19 does not constitute experience of a traumatic event per se, the PDI was selected for the purpose of implementing a previously developed measure of emotional distress and physiological arousal secondary to ongoing or recent events (55). As such, this modified measure provided a structured assessment of distress attributable to the ongoing pandemic. Previous reports on the PDI have demonstrated good internal consistency, test-retest reliability, and convergent and divergent validity of the measure (55). Internal consistency of the current sample was 0.94. Consistent with past research, the overall score on this measure is the mean response across all 13 items.

#### Alcohol Consumption

Indices of recent alcohol use were assessed with the National Institutes on Alcohol Abuse and Alcoholism (NIAAA) Recommended Alcohol Questions^1^. The items are as follows: (1) “how often did you usually have any kind of drink containing alcohol?”; (2) “how many alcoholic drinks did you have on a typical day when you drank alcohol?”; (3) “what is the largest number of drinks containing alcohol that you drank within a 24-h period?”; (4) “how often did you drink this largest number of drinks?,” and; (5) “how often did you have 5 or more (males) or 4 or more (females) drinks containing alcohol within a 2-h period.” The latter item provides the operational definition of a “binge” drinking episode used in the present study. An additional item was included to query the amount of time participants typically spend consuming alcohol per day, with options ranging from 1 (0 h) to 7 (10+ h).

#### Solitary Drinking Frequency

Questions on drinking context were adapted from those reported in Keough et al. (24). These questions were originally adapted from Cooper's (56) drinking contexts measure. A single item was used to assess relative frequency of solitary drinking in the specified 1 month period: “when you drank alcohol, how much of that time was spent drinking while you were by yourself relative to when socializing with other people either in-person or virtually.” Response options ranged from 1 (“100% by yourself”), 2 (90% by yourself, 10% with other people) to 10 (10% by yourself, 90% with other people”), 11 (“100% with other people”). An additional item was used to assess relative frequency of social drinking in in-person relative to virtual contexts: “when you drank alcohol while socializing with other people, how much of that time was spent with other people in-person relative to being virtually.” Response options ranged from 1 (100% in person), 2 (90% in person, 10% virtual) to 10 (10% in person, 90% virtual), 11 (100% virtual).

#### Alcohol Purchase Task (APT)

The APT is a hypothetical commodity purchase task that provides quantitative indices of demand for alcohol (57). Participants were asked to indicate how many drinks they would consume at the following prices: $0, $0.50, $1.00, $1.50, $2.00, $2.50, $3.00, $4.00, $5.00, $6.00, $7.00, $8.00, $9.00, $10.00, $11.00, $12.00, $13.00, $14.00, and $15.00. Participants were instructed that all drinks were administered as “standard” sizes (equivalent to one standard drink), that they could not stockpile drinks for a later time (i.e., all requested drinks must be consumed), and that they did not drink before and cannot drink after [adapted from (58)]. Five scores can be generated from the APT that reflect the latent facets of alcohol demand: intensity (consumption when alcohol is free); breakpoint (the first price that reduces alcohol consumption to 0); Omax (maximum expenditure for alcohol); Pmax (the price associated with the maximum expenditure), and elasticity (sensitivity of consumption across increasing prices of alcohol) (57). Test-retest reliability of the scores of the APT have been previously reported to range between *r* = 0.58 to *r* = 0.91, depending on the index being scored (59). The APT has also demonstrated predictive validity for the quantity of drinks consumed among college students at 1-month follow-up and alcohol problems at 6-month follow-up (60). Convergent validity has also been demonstrated between the APT and self-report measures of drinking quantity and alcohol related problems (27).

Nonsystematic APT data were identified using a 3-criterion algorithm proposed by Stein et al. (61). Briefly, this algorithm detects cases that violate the trend (non-negligible reduction in consumption as price increases), bounce (less than a 10% incidence of local price-to-price increases in consumption), and reversals from zero (non-zero consumption following two consecutive zero consumption) criteria. Benchmarks (i.e., cases with <0.025 log-unit reductions in consumption across prices; >10% incidence of bounce, and; any reversals from zero) were implemented as described by Stein et al. (61). Any cases where at least one of these criteria were violated (for pre- or post-social distancing) were excluded from APT analyses. Freely available scoring software in R (“beezdemand”) was used to estimate the observed values of intensity, breakpoint, OMax, and Pmax as well as the derived value for elasticity across prices (62). Indices of demand were derived using the exponentiated approach, as outlined by Koffarnus et al. (63).

#### Drinking Motives Questionnaire—Revised (DMQ-R)

The DMQ-R is a 20-item questionnaire that assesses motives to consume alcohol (56). Items are scored on a 5-point scale from 1 (*almost never*) to 5 (*almost always/always*). The measure has 4 subscales: social (e.g., “Because it helps you enjoy a party”), coping (e.g., “To forget your worries”), conformity (e.g., “Because your friends pressure you to drink”), and enhancement [e.g., “Because it gives you a pleasant feeling”; (56)]. In the present study, responses were anchored to a 30-day timeframe. Of primary interest was the coping subscale score, however, all 4 subscales were scored and included in statistical models (as described in Analytical Plan). The DMQ-R has demonstrated good to excellent test-retest reliability, internal consistency, and predictive validity for concurrent drinking frequency and quantity and alcohol-related problems among a sample of undergraduate students (37). Internal consistency of the four DMQ subscales in the current sample ranged from 0.84 to 0.95 across both assessed timeframes.

#### Patient Health Questionnaire (PHQ)-9

The PHQ-9 is a widely used 9-item self-report measure of depression severity (64). Participants are asked to rate how often they are bothered by the specific item, ranging from 0 (*not at all*) to 3 (*nearly every day*). To address the aims of this study we adjusted the instructional set to assess a 30-day timeframe, rather than the traditional 14-day timeframe. A single severity score for each timeframe, derived by summing responses to all 9 items, was used as the primary outcome (64). A systematic review of the PHQ-9 has reported sound psychometric properties of the measure, including internal reliability, test-retest reliability, and convergent validity with other measures of depression (65). Internal consistency in the current sample was 0.94 and 0.93 for the pre- and post-social distancing timeframes, respectively.

#### Reward Probability Index (RPI)

The RPI is a self-report scale designed to measure the availability of response-contingent positive reinforcement (reward probability) as well as the presence of aversive stimuli (environmental suppressors) in an individual's environment (66). The RPI accomplishes this with a 20-item scale scored on a 4-point Likert scale from 1 (*strongly disagree*) to 4 (*strongly agree*). Two subscale scores can be derived: reward probability (e.g., “I feel a strong sense of achievement”) and environmental suppressors (e.g., “Changes have happened in my life that have made it hard to find enjoyment”). Subscale scores are obtained by summing the scores on 10 constituent items. The 10 items that contribute to the environmental suppressors subscale are reverse scored before being summed. As such, higher scores on these two subscales represent greater reward probability and fewer environmental suppressors, respectively. A single total score was also obtained by summing the two subscale scores. Higher scores on this aggregate score represent both increased access to environmental reward and decreased presence of environmental suppressors. The RPI has previously demonstrated high internal consistency, test-retest reliability, convergent validity and discriminant validity (66). Internal consistency for the total scale in the current sample was 0.90 and 0.88 for the pre- and post-social distancing timeframes, respectively.

#### COVID-19 Impact and Perception

For descriptive purposes, questions were developed to estimate the impact of COVID-19 on individuals' income and employment; participants were provided with 8 response options ranging from 1 (“*My income/employment has increased*”) to 8 (“*I have lost 100% of my income/employment*”). Similarly, a non-standardized question assessing worry secondary to COVID-19 was included where participants were asked to indicate how worried they are about COVID-19 ranging from 1 (“*not worried at all*”) to 7 (“*extremely worried*”). These outcomes were included to illustrate the sample characteristics and impact of COVID-19 specifically.

### Analysis Plan

Prior to analysis, all variables were assessed for univariate normality and the presence of outliers. All variables were normally distributed. Univariate outliers were defined as data points that fell outside of ±3.29 SD of the mean. Outliers were only observed on the RPI scale and APT. These outliers were deemed to be valid points of data but were nonetheless winsorized to ±3.29 SD to reduce their extreme influence on analyses (67). Multiple imputation was used to address missing data (assumed missing at random).

To address aim 1 of examining self-reported differences in outcomes as a function of timeframe (pre- and post-social distancing), paired samples *t*-tests were conducted to determine whether observed scores on the specified outcomes of interest were significantly different post-social-distancing compared to pre-social-distancing. Independent samples *t*-tests were then conducted to determine whether any of the specified outcomes differed as a function of race (as an exploratory analysis). Because our sample was predominantly white (65.5%), we computed a binary variable to compare white participants with non-white participants for the pairwise comparisons.

To address aim 2 of assessing the indirect effect of environmental reward and psychological distress on alcohol consumption through depression and coping motives, an index of alcohol consumption was derived by taking the product of typical alcohol consumption frequency and quantity, NIAAA recommended questions 1 and 2 (68). This index (“alcohol QF”) was derived for both pre-social-distancing and post-social-distancing timeframes; higher scores on this index are indicative of greater levels of alcohol consumption. The post-social-distancing alcohol QF score served as our primary outcome in our mediation models. However, because we observed significant pre-social-distancing to post-social-distancing differences in frequency of binge drinking and frequency of solitary drinking, we ran additional exploratory models with these specified as the outcome of interest.

To test the mediation hypotheses, mediation effects were examined using Hayes' (69) PROCESS macro for SPSS. To address the first hypothesis, we modeled the indirect effect of post-social-distancing environmental reward (RPI total score) on post-social-distancing alcohol QF through post-social-distancing depression severity (PHQ) and post-social-distancing coping motives (DMQ-R coping motive subscale). We included pre-social-distancing alcohol QF, depression, and coping motives as covariates in the mediation model in order to examine associations among post-social-distancing variables *relative to* pre-social-distancing levels. To assess hypothesis 2, we modeled the indirect effect of COVID-19-related distress (PDI) on post-social-distancing alcohol QF through post-social-distancing coping motives. Consistent with hypothesis 1, we included pre-social-distancing alcohol QF and coping motives as covariates. Pre-social-distancing social, enhancement, and conformity motive scores (DMQ-R) were included as covariates in all mediation models. This facilitated the examination of the unique role of coping motives as a mediator. Sex and race were also included as covariates in all mediation models. A mediation effect was deemed to be significant if the indirect effect's 95% bias-corrected bootstrap confidence interval did not contain 0.

## Results

### Sample and Demographics

After screening for eligibility and agreement to participate, 1,127 participants proceeded to the survey. Of the 727 participants who were excluded, 5 participants did not agree to participate after reading the information statement and 722 did not meet one or more eligibility criteria. After screening for inattention, 833 cases were retained for analyses. The final sample was mostly male (64.7%) with an average age of 40.76 (SD = 10.65) years. Reported racial backgrounds included White (65.5%); Black or African American (14.9%); Asian or Asian American (6.7%); Hispanic or Latino (6.2%); Alaska Native or American Indian (0.6%); Native Hawaiian or other Pacific Island (0.1%), or more than one identified racial background (1.8%). Most participants were not students (61%) and reported an average household income of $50,000-$70,000 per year. On average, participants reported living with 2.37 others (66.1% with family). Table 1 provides a summary of additional sample characteristics.

**Table 1:** Sample Characteristics.

	**M (SD)/%**
AUDIT Total score—past year (*SD*)	10.49 (8.13)
Frequency of past-year drinking	
Every day	15.80%
5 to 6 times per week	16.30%
3 to 4 times per week	24.60%
twice a week	22.40%
once a week	13.00%
2 to 3 times per month	7.80%
Living arrangement	
With family	66.1%
Live alone	22.9%
With roommate	8.6%
Other	1.9%
Number of residents in household (*SD*)	2.37 (1.49)
Income change due to COVID	
Increased	4.3%
No change	41.7%
Reduced up to 10%	12.7%
Reduced by 10–25%	19.1%
Reduced by 25–50%	11.4%
Reduced by 51–75%	4.2%
Reduced by more than 75%	2.0%
100% income loss	4.3%
Change in hours working due to COVID	
Working the same # hours	43.9%
Working more hours	13.2%
Working fewer hours	34.2%
On leave, terminated or quit	8.5%
COVID-related worry (*SD*)	4.69 (1.67)
PDI Total Score - anchored to COVID (*SD*)	1.19 (0.93)

*AUDIT, Alcohol use disorders identification test. PDI, Peritraumatic Distress Inventory (anchored to COVID-19)*.

### Self-Reported Change (Pre-social-distancing vs. Post-social-distancing) in Primary Outcomes

Pairwise comparisons of pre-social-distancing and post-social-distancing outcomes are presented in Table 2. A conservative Bonferroni correction was applied to mitigate false positive findings in the context of multiple comparisons. Findings were interpreted as significant at a threshold of *p* < 0.002. Consistent with hypotheses, participants reported greater severity of depressive symptoms post-social-distancing, as well as reported lower total RPI score post-social-distancing. Overall, participants reported typical quantities, frequency, and time spent drinking (NIAAA item 1 and 2) post-social-distancing that were commensurate with pre-social-distancing values. However, participants reported significantly more binge episodes post-social-distancing. As predicted, participants endorsed significantly higher coping motives post-social-distancing compared to pre-social-distancing. Conversely, participants endorsed significantly lower social, conformity, and enhancement motives for drinking post-social-distancing relative to pre-social-distancing. Additionally, participants reported significantly more frequent solitary drinking (but also more virtual social drinking) post-social-distancing compared to pre-social-distancing.

**Table 2:** Paired Samples *t*-test Statistics.

**Outcome**	**Pre-social-distancing *M* (*SD*)**	**Post-social-distancing *M (SD)***	***t***	***p***
Alcohol QF	17.35 (14.45)	17.38 (13.83)	−0.070	0.944
NIAAA: Frequency	5.19 (1.59)	5.18 (1.79)^†^	0.231	0.818
NIAAA: Quantity	3.16 (2.09)^‡^	3.15 (1.95)	0.075	0.940
NIAAA: Time	2.63 (0.94)	2.69 (1.08)	−2.039	0.042
NIAAA: Max drinks	4.02 (1.94)	3.95 (2.01)	1.560	0.119
NIAAA: Binge frequency	2.85 (1.97)^‡^	3.00 (2.03)	−3.220	**0.001**
RPI: Reward probability	33.95 (5.68)	30.23 (6.60)	18.823	**<0.001**
RPI: Environmental suppressors	26.53 (6.82)^†^	25.67 (6.53)^†^	6.088	**<0.001**
RPI: Total	60.40 (9.94)^†^	55.89 (9.61)	16.771	**<0.001**
DMQ: Social motives	2.71 (1.12)^‡^	2.08 (1.20)^‡^	19.239	**<0.001**
DMQ: Coping motives	2.38 (1.09)^‡^	2.49 (1.12)^‡^	−5.356	**<0.001**
DMQ: Enhancement motives	2.82 (1.00)^‡^	2.73 (1.03)^‡^	4.095	**<0.001**
DMQ: Conformity motives	1.91 (1.10)^‡^	1.79 (1.13)^‡^	6.507	**<0.001**
PHQ: Total	6.58 (6.99)^‡^	7.49 (7.01)^‡^	−7.683	**<0.001**
Solitary drinking frequency	6.73 (3.29)	5.14 (3.52)	16.169	**<0.001**
Virtual drinking frequency	3.83 (3.42)^‡^	5.36 (3.91)^‡^	−12.188	**<0.001**
APT: Intensity	8.61 (27.74)	7.34 (10.23)	1.425	0.155
APT: Breakpoint	9.89 (4.54)^‡^	9.72 (4.65)	2.245	0.025
APT: Omax	21.43 (22.04)	23.93 (27.55)	−4.624	**<0.001**
APT: Pmax	7.25 (3.88)	7.10 (3.79)	1.441	0.150
APT: Elasticity	0.026 (0.21)	0.076 (1.04)	−1.509	0.132

*NIAAA, National Institute on Alcohol Abuse and Alcoholism (questions from 5-item set); RPI, Reward Probability Index; Items that contribute to the environmental suppressors subscale of the Reward Probability Index are reverse-scored. DMQ, Drinking Motives Questionnaire; APT, Alcohol Purchase Task; Statistical significance threshold set at 0.002 to correct for family wise error*.

† *Mean score for white participants significantly greater than non-white mean score (p < 0.002)*.

‡ *Mean score for non-white participants significantly greater than white mean score (p < 0.002)*.

*Bolded values p < 0.002*.

Screening of data for the APT resulted in a final sample of 629 cases with valid pre-and post-social-distancing data for alcohol demand. Results for alcohol demand varied by demand index. Intensity of demand, elasticity across prices, breakpoint, and price associated with maximum expenditure (Pmax) did not differ from pre- to post-social-distancing. Together, these suggest that alcohol consumption at no cost, sensitivity of alcohol consumption to increases in price, price associated with zero consumption, and the point at which individual demand transitions from inelastic to elastic, respectively, were consistent across timeframes. Maximum expenditure was found to be higher post-social-distancing compared to pre-social distancing. Increased expenditure suggests that participants had a higher maximal response output post-social-distancing compared to pre-social distancing. Together these results might suggest there are subtle changes to some facets of alcohol demand.

The results of the independent samples *t*-tests to explore differences in outcome as a function of race are presented in Table 2 (see footnote). Non-white participants reported less frequent alcohol consumption post-social-distancing and greater typical quantity of alcohol consumed pre-social-distancing. At pre-social-distancing, non-white participants reported higher frequency of binge consumption, greater environmental suppression, and reduced environmental reward probability relative to white participants. Non-white participants also reported greater environmental suppression post-social-distancing and higher endorsement for all drinking motives subscales at both timepoints. Not reported in the table, non-white participants reported higher levels of COVID-19-related distress and greater severity of depressive symptoms at both timepoints (*p* < 0.002 for both outcomes). Finally, non-white participants scored higher on one index of alcohol demand (breakpoint) at pre-social-distancing. The remaining measures did not differ by race (all *p* > 0.002).

### Indirect Association of Environmental Reward With Alcohol Use via Severity of Depressive Symptoms and Coping Motives

**Figure 1:**
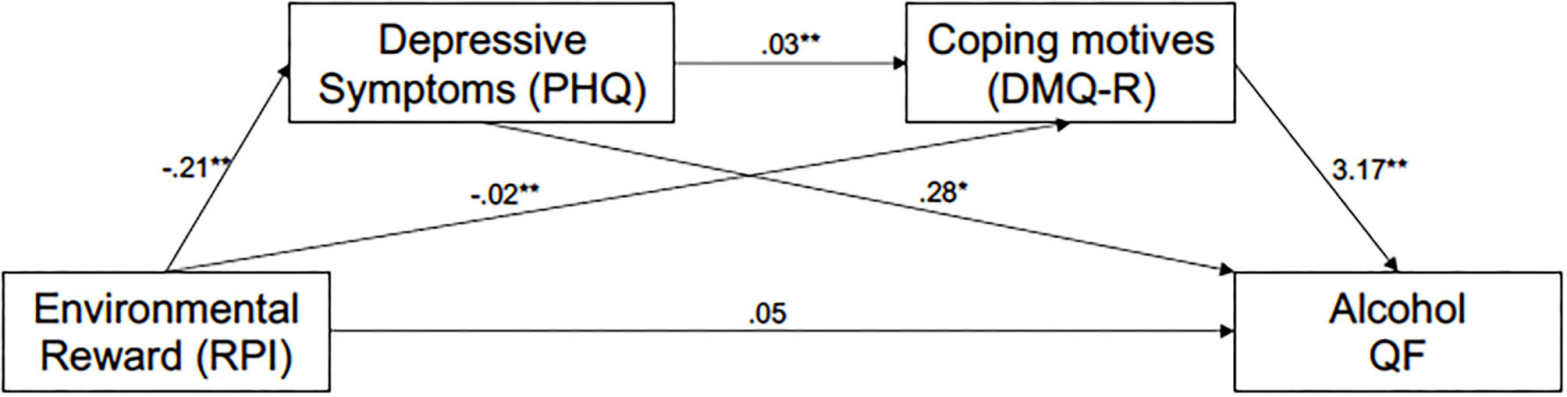
Sequential indirect effect of environmental reward on alcohol QF through severity of depressive symptoms and coping motives. *p < 0.05, **p < 0.01. RPI, Reward Probability Index; PHQ, Patient Health Questionnaire; DMQ-R, Drinking Motives Questionnaire—Revised; Alcohol QF, measure of alcohol quantity/frequency (see Analysis Plan). All variables shown in the model correspond to post-social-distancing scores. Environmental reward indirectly significantly predicted alcohol QF through three unique paths: sequentially through depressive symptoms then coping motives; coping motives only, and; depressive symptoms only. Path coefficients are unstandardized *b* values. Sex and race were included as demographic covariates. Pre-social-distancing covariates included: environmental reward probability, depressive symptoms, motives (coping, enhancement, conformity, social), and alcohol QF.

A summary of the direct and indirect effects for all mediation models conducted in the study can be found in Table 3. The results of the sequential mediation model examining the indirect effect of post-social-distancing environmental reward on post-social-distancing alcohol QF through severity of post-social-distancing depressive symptoms and post-social-distancing coping motives (controlling for pre-social-distancing values) are presented in Figure 1. There was a significant indirect effect of environmental reward (total RPI score) on alcohol QF via severity of depressive symptoms and coping motives. Specifically, lower levels of reward probability predicted greater severity of depressive symptoms; greater severity of depressive symptoms, in turn, predicted higher levels of coping motives; higher levels of coping motives subsequently predicted increases in alcohol QF. Significant unique indirect effects of total RPI score with alcohol QF were also observed through depression and coping motives, in the directions specified above. There was no significant direct effect of environmental reward on alcohol QF. Regarding covariates, race (*b* = −1.727, *SE* = 0.684, *t* = −2.523, *p* = 0.012), pre-social distancing coping motives (*b* = −1.295, *SE* = 0.649, *t* = −1.995, *p* = 0.046), pre-social distancing enhancement motives (*b* = 0.959, *SE* = 0.454, *t* = 2.110, *p* = 0.035), and pre-social distancing alcohol QF (*b* = 0.656, *SE* = 0.023, *t* = 27.858, *p* < 0.0001) all significantly predicted variance in the post-social-distancing alcohol QF outcome. No other covariates were statistically significant predictors of post-social-distancing alcohol QF (all *p* > 0.05).

**Table 3:** Indirect and Direct Effects for hypothesized mediation models.

	**Outcome: Post-social-distancing alcohol QF**					
	***b***	***SE b***	***LLCI***	***ULCI***	***t***	***p***
Mediation Model 1						
Direct Effect (Reward Probability Index)	0.051	0.051			1.011	0.312
Indirect Effects						
Depression severity (PHQ)	−0.060	0.024	−0.108	−0.014		
Coping motives (DMQ-R)	−0.054	0.015	−0.087	−0.027		
Sequential effect	−0.024	0.008	−0.041	−0.010		
Mediation Model 2						
Direct Effect (COVID-related distress)	0.901	0.503			1.789	0.074
Indirect Effect (Coping motives)	0.805	0.209	0.436	1.256		

*Mediation model 1: indirect effect of environmental reward on alcohol use sequentially through severity of depressive symptoms and coping motives. PHQ, Patient Health Questionnaire; DMQ-R, Drinking Motives Questionnaire—Revised. Mediation model 2: indirect effect of COVID-related distress on alcohol use through coping motives. Confidence intervals presented here are 95% bias-corrected bootstrap estimates*.

Results of the exploratory sequential mediation analysis with frequency of binge drinking specified as the outcome were consistent with the primary model. There was a significant indirect sequential effect of environmental reward on binge frequency through severity of depression and coping motives (*b* = −0.003, *SE* = 0.001, 95% bootstrap CI [−0.005, −0.001]). There was also a unique indirect effect of environmental reward on binge frequency through coping motives (*b* = −0.007, *SE* = 0.002, 95% bootstrap CI [−0.011, −0.003]) but not through depressive symptoms (*b* = −0.007, 0.004, 95% bootstrap CI [−0.014, 0.001]). Consistent with the first model, there was no direct effect of environmental reward on binge frequency (*p* > 0.05). Conversely, there were no significant indirect effects in the exploratory model with frequency of solitary drinking specified as the primary outcome (all bootstrap CIs contained zero). However, there was a significant direct effect of environmental reward on frequency of solitary drinking (*b* = 0.082, *SE* = 0.015, *t* = 5.585, *p* < 0.0001).

### Indirect Association of COVID-19-Related Distress With Alcohol Use via Coping Motives

**Figure 2:**
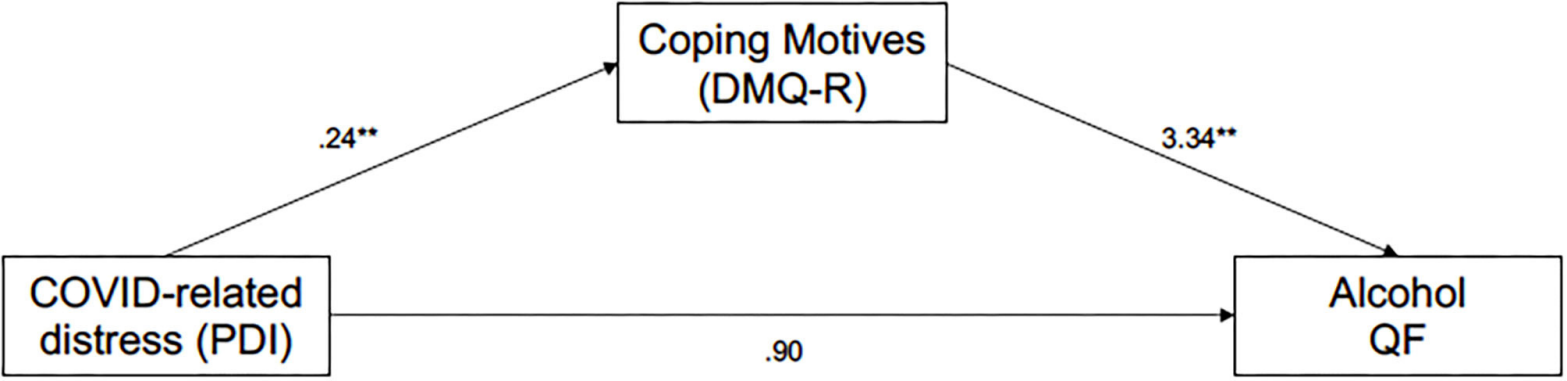
Indirect effect of COVID-related distress on alcohol QF through coping motives. **p < 0.01. PDI, Peritraumatic Distress Inventory; DMQ-R, Drinking Motives Questionnaire—Revised; Alcohol QF, measure of alcohol quantity/frequency (see Analysis Plan). Post-social-distancing coping motives and post-social-distancing alcohol QF are shown in the model. COVID-related distress was not anchored to a specific timeframe. The indirect effect of COVID-related distress on alcohol QF through coping motives was significant. Path coefficients are unstandardized *b* values. Sex and race were included as demographic covariates. Pre-social-distancing covariates included: motives (coping, enhancement, conformity, social), and alcohol QF.

The results of the mediation model examining the indirect effect of COVID-19-related distress on typical alcohol consumption quantity and frequency through coping motives (controlling for pre-social-distancing values) are presented in Figure 2. There was a significant indirect effect of post-social-distancing COVID-19-related distress on post-social-distancing alcohol QF through coping motives (Table 3). Specifically, higher levels of COVID-19-related distress predicted greater levels of drinking to cope that, in turn, predicted greater alcohol QF. The direct effect of COVID-19-related distress on alcohol QF was not significant, suggesting a full mediation of the effect. Regarding covariates, pre-social-distancing enhancement motives (*b* = 0.960, *SE* = 0.451, *t* = 2.127, *p* = 0.034), race (*b* = −1.616, *SE* = 0.679, *t* = −2.379, *p* = 0.018), and pre-social-distancing alcohol QF (*b* = 0.659, *SE* = 0.024, *t* = 28.11, *p* < 0.0001) significantly predicted post-social-distancing alcohol QF. None of the other covariates reached the threshold of statistical significance (all *p* > 0.05).

For the exploratory analyses examining binge and solitary drinking, we first conducted the mediation analysis with post-social-distancing frequency of binge drinking specified as the outcome. There was a significant indirect effect of COVID-related distress on binge frequency through coping motives (*b* = 0.093, *SE* = 0.029, 95% bootstrap CI [0.043, 0.155]). The direct effect of COVID-related distress on binge frequency was not significant (*p* > 0.05) suggesting a full mediation of the effect. Finally, we ran the mediation analysis with post-social-distancing with frequency of solitary drinking specified as the outcome. There was a significant indirect effect of COVID-related distress on frequency of solitary drinking through coping motives (*b* = −0.090, *SE* = 0.048, 95% bootstrap CI [−0.189, −0.001]). Specifically, greater levels of COVID-related distress predicted higher levels of coping motives, which in turn predicted greater frequency of solitary drinking. There was no significant direct effect of COVID-related distress on solitary drinking frequency (*p* > 0.05) suggesting a full mediation of the effect.

## Discussion

The primary aims of this study were to estimate self-reported changes in alcohol consumption, depression, environmental reward and drinking motives during COVID-19, and to test theoretically based mediation models involving these outcomes. Regarding the first aim, we observed inconsistency in the magnitude and direction of self-reported change across alcohol measures. For example, participants reported a greater frequency of binge drinking, but no change in the quantity and frequency of alcohol use. Self-reported changes in alcohol demand indices were also variable, with some indices suggesting no change (e.g., intensity, elasticity) and others supporting change (e.g., maximum expenditure). Overall, however, these results are consistent with predictions of individual differences in the presence and direction of changes in alcohol use (4) and suggest variability in the presence and magnitude of alcohol use indices in the context of social distancing related to COVID-19 [e.g., (11)].

We also found evidence of greater severity of depressive symptoms, lower levels of environmental reward and higher levels of environmental suppressors post-social-distancing compared to pre-social-distancing. These findings are in keeping with the behavioral theory of depression suggesting that restrictions in access to environmental and social rewards increase risk of depression [e.g., (16)], and with past research that documented an increased incidence of depression in individuals quarantined during the SARS epidemic (21). We also found that self-reported frequency of negative reinforcement drinking motivated by internal contexts (i.e., coping) increased from pre- to post-social-distancing timeframes, as hypothesized. Conversely, positive reinforcement drinking motives (i.e., enhancement, social) and negative reinforcement motives related to external social contexts (i.e., conformity) decreased post-social-distancing relative to pre-social-distancing. Contextual factors surrounding COVID-19 (i.e., social distancing) may contribute to these observed changes in motivations for alcohol consumptions. It is intuitive that externally-motivated reasons for drinking might decrease during periods of social distancing. Similarly, greater negative reinforcement motives for drinking are intuitive in the context of observed higher negative affect observed post-social-distancing compared to pre-social-distancing.

Notably, exploratory analyses showed that race was significantly associated with many of our predictors (environmental reward, depressive symptoms, motives) and some alcohol use outcomes. Generally, non-white participants seemed to be at higher risk for higher drinking levels, riskier drinking patterns, and greater affective distress, when compared to white participants. Because we did not design our study to examine race- and demographic-based differences (e.g., we did not comprehensively assess socioeconomic status), we cannot make meaningful inferences about these differences. Moreover, the aggregation of non-White participants into a single group precludes the examination of differences between non-white groups and limits any nuanced conclusions concerning the association of race with the outcomes reported here. Nonetheless, these data are in keeping with predicted disparities in mental health outcomes for marginalized groups [e.g., (70)] and are consistent with reports of racial and ethnic-based health disparities during the COVID-19 pandemic (71). Ultimately, the data reported here emphasize the need for additional research to more closely examine how race and other demographic factors have impacted and will continue to impact individuals' response to the COVID-19 pandemic and associated mental health outcomes.

In addition to examining mean-level differences, we also tested theory-based mediation models to examine predictors of alcohol use during the COVID-19 pandemic. In the context of behavioral theory of depression and self-medication theories, the first set of mediation models tested environmental reward as an indirect predictor of alcohol QF post-social-distancing through severity of depressive symptoms and drinking to cope. Results suggested that: (1) lower levels of environmental reward predicted greater severity of depressive symptoms; (2) greater severity of depressive symptoms predicted higher levels of coping motives, and; (3) higher coping motives, in turn, predicted greater levels of alcohol consumption. In addition to the total sequential mediation effect, both severity of depressive symptoms and drinking to cope also independently mediated the effect between environmental reward and alcohol QF. The second mediation model, derived from the self-medication model, examined coping motives as a mediator of the relationship between COVID-19-related distress (secondary to COVID-19) and alcohol use post-social-distancing. The data supported our hypothesis for both typical alcohol QF: (1) higher levels of COVID-19-related distress predicted greater levels of coping motives that, in turn; (2) predicted higher levels of alcohol use post-social-distancing. In exploratory analyses, the results of the two mediation models replicated using post-social-distancing frequency of binge drinking at the primary outcome. Collectively these results are generally consistent with the behavioral theory of depression and self-medication hypothesis, where restrictions in environmental reward predict increases in the severity of depression (15, 16) and drinking to cope is hypothesized to mediate the relationship between negative affect (e.g., depression) and alcohol use (32, 33).

Because coping motives only partially mediated the hypothesized effects, it remains likely that other factors not assessed here also predict relative change in alcohol use during the COVID-19 pandemic. For example, we found that pre-social-distancing enhancement motives (i.e., drinking to enhance positive states) was significantly related to both alcohol consumption and frequency of binge drinking. While this finding was somewhat unanticipated, enhancement motives are typically strong predictors of alcohol consumption (31). Exactly how changes in motives—and other constructs—predict relative change in alcohol use during the COVID-19 pandemic is an important question for future research.

Other theoretical frameworks, notably behavioral theories of choice, might also provide insight into the data presented here. For example, behavioral economic theories of substance use disorders posit that the decision to use or abstain from a drug is the result of a joint influence of internal motivational states and availability of alternative reinforcers in the environment (72). Human research provides confirmatory evidence of the inverse relationship between availability of alternate reinforcers and alcohol use/problems [e.g., (73–75)]. In the context of social-distancing due to the COVID-19 pandemic, it is possible that changes in alcohol use and alcohol demand are directly influenced by changes in availability of alternative reinforcers. However, as highlighted in a recent review the RPI does not explicitly measure substance-free reinforcement (76) and so the present data cannot be parsimoniously interpreted within choice theory frameworks. Nonetheless, behavioral theories of choice are likely well-suited to studying the effects of mandated social distancing on substance use and in reconciling the discordant results of changes in alcohol demand reported here. Future research based on such theories is warranted.

In our additional exploratory models, we also found that frequency of solitary drinking post-social-distancing was not predicted by COVID-related distress or depressive symptoms. We did, however, observe a significant indirect effect of COVID-related distress on solitary drinking frequency through coping motives, and a direct effect of environmental reward on solitary drinking frequency post-social-distancing. Regarding the latter, lower levels of post-social-distancing environmental reward were associated with greater frequency of solitary drinking. This pattern of results suggest that differences in external contextual factors, in addition to select internal context factors (i.e., COVID-19 related distress through alcohol motives), are relevant for predicting solitary drinking in the context of COVID-19 emergency measures. This finding is in partial agreement with other recent research examining alcohol use in the context of COVID-19. Specifically, in another study of alcohol use during the early stages of the pandemic, living alone (an external context factor) predicted increased solitary drinking, whereas internal context factors did not (77). Because past research has demonstrated that increased frequency of solitary drinking predicts increases in alcohol-related problems (24, 78) it is imperative to understand which specific environmental factors during COVID-19 may elevate individuals' risk to develop this pattern of drinking.

Ultimately, one significant implication of these findings is to highlight factors that may be associated with risk for elevated rates of alcohol use disorders during the pandemic. Such risk factors include, for example, the elevated frequency of binge drinking and solitary use of alcohol post-social distancing compared to pre-social distancing. Both binge drinking and the use of alcohol in solitary contexts are considered risky patterns of alcohol use, in part due to their relation to future alcohol-related problems [e.g., (24, 79)] To the extent that interventions mitigate constrained environmental reward secondary to social distancing, they might have beneficial effects in preventing escalations in alcohol consumption and the increased frequency of risky drinking patterns. Behavioral activation (BA) interventions represent an appealing option, as they are effective in targeting both depressive symptoms and substance use (80). Moreover, such interventions can effectively be delivered remotely, via smartphone technology, enhancing the potential utility to a broader population [e.g., (81)]. In the context of COVID-19, the implementation of a BA-oriented intervention is therefore not only theoretically justified, but has the potential for feasible wide-spread implementation. Smartphone-based interventions that incorporate coping-skills training, psychoeducation, and related interventions have also been developed [e.g., (82)] that might be useful as adjunct therapy for individuals whose changes in alcohol use are driven by coping-related motives. Future research will be required to determine the efficacy and utility of such interventions in these contexts.

There are key limitations of the present research that should be noted. First, our research employed a cross-sectional approach that required participants to selectively report on two distinct timeframes, which may introduce bias. For example, a negative retrieval bias may selectively enhance accessibility of negatively-valanced events for some individuals [e.g., (83)]. Similarly, simple demand characteristics of the questionnaire due to the timeframe instructional set (i.e., anchor of items batteries to pre-social-distancing and post-social-distancing) might provoke unintentional bias and unduly influence individuals' responses (84). Second, the use of a cross-sectional approach precludes any inference about causality or changes over time. Third, sample representativeness and participant eligibility criteria restricts generalization of results to the general population. For example, we selected participants based on a minimum frequency of past-year drinking history and an age >21 years old. As a result, we cannot extend conclusions about findings from this sample to individuals with less frequent patterns of drinking, those abstaining from alcohol, underage drinkers, those with remote histories of alcohol use, or alcohol naïve individuals who started drinking during the pandemic or immediately prior. More broadly, our use of a convenience sample from MTurk limits generalizations to the general population due to sample representativeness. Future research will be essential to address these limitations to confirm the replicability and generalizability of the findings reported here.

Despite these limitations, our results provide initial evidence for factors related to changes in alcohol consumption during COVID-19. Some results are consistent with predictions of increased incidence of alcohol use disorders following the easement of social distancing procedures, at least in certain vulnerable subgroups [e.g., (10)]. Such knowledge can inform public health initiatives to curb harmful use of alcohol and may provide clinicians with useful knowledge concerning both risk and protective factors for alcohol use during the present, and future, pandemics. Prospective research will be needed to replicate the results reported here, and to establish the long-term consequences of these changes observed during the COVID-19 pandemic.

## Data Availability Statement

The raw data supporting the conclusions of this article will be made available by the authors, without undue reservation.

## Ethics Statement

The studies involving human participants were reviewed and approved by University of Toronto Health Sciences Research Ethics Board. All participants read an informational statement and indicated agreement to participate in the study in accordance with institutional requirements and national legislation.

## Author Contributions

MM and CH conceived the idea for the research project and developed the primary hypotheses tested in this study. MM, CH, SR, LH, and MK contributed to the design of the study protocol. MM and SR completed data collection under the supervision of CH. MM, CH, MK, and JW contributed to statistical analyses and interpretations of data. MM, CH, and SR contributed to writing drafts of the manuscript. MM, CH, SR, LH, MK, and JW provided feedback on drafts of the manuscript and approved the final version. All authors contributed to the article and approved the submitted version.

## Conflict of Interest

The authors declare that the research was conducted in the absence of any commercial or financial relationships that could be construed as a potential conflict of interest.

## Footnotes

^1^https://www.niaaa.nih.gov/research/guidelines-and-resources/recommended-alcohol-questions
